# Practitioner Perspectives on the Uses of Generative AI Chatbots in Mental Health Care: Mixed Methods Study

**DOI:** 10.2196/71065

**Published:** 2025-09-16

**Authors:** Jessie Goldie, Simon Dennis, Lyndsey Hipgrave, Amanda Coleman

**Affiliations:** 1 Melbourne School of Psychological Sciences, Medicine, Dentistry and Health Sciences, University of Melbourne, Grattan Street, Parkville, Melbourne, 3010, Australia, 61 467607835; 2 Monash School of Psychological Sciences, Faculty of Medicine, Nursing and Health Sciences, Monash University, Melbourne, Australia

**Keywords:** artificial intelligence, ChatGPT, mental health care, practitioner perspectives, mixed methods, digital health

## Abstract

**Background:**

Generative artificial intelligence (AI) chatbots have the potential to improve mental health care for practitioners and clients. Evidence demonstrates that AI chatbots can assist with tasks such as documentation, research, counseling, and therapeutic exercises. However, research examining practitioners’ perspectives is limited.

**Objective:**

This mixed-methods study investigates: (1) practitioners’ perspectives on different uses of generative AI chatbots; (2) their likelihood of recommending chatbots to clients; and (3) whether recommendation likelihood increases after viewing a demonstration.

**Methods:**

Participants were 23 mental health practitioners, including 17 females and 6 males, with a mean age of 39.39 (SD 16.20) years. In 45-minute interviews, participants selected their 3 most helpful uses of chatbots from 11 options and rated their likelihood of recommending chatbots to clients on a Likert scale before and after an 11-minute chatbot demonstration.

**Results:**

Binomial tests found that Generating case notes was selected at greater-than-chance levels ( 15/23, 65%; *P*=.001), while Support with session planning (*P*=.86) and Identifying and suggesting literature (*P*=.10) were not. Although 55% (12/23) were likely to recommend chatbots to clients, a binomial test found no significant difference from the 50% threshold (*P*=.74). A paired samples *t* test found that recommendation likelihood increased significantly (19/23, 83%; *P*=.002) from predemonstration to postdemonstration.

**Conclusions:**

Findings suggest practitioners favor administrative uses of generative AI and are more likely to recommend chatbots to clients after exposure. This study highlights a need for practitioner education and guidelines to support safe and effective AI integration in mental health care.

## Introduction

### Overview

Globally, the prevalence of mental illness is rising and health care systems are under increasing pressure [1]. In Australia, individuals face barriers to mental health care, including financial costs and extended wait times for appointments, while practitioners are struggling to meet demand [2,3]. In this context, artificial intelligence (AI) is a potential game changer, offering 24/7 support for individuals, thus alleviating pressure on mental health practitioners [4,5]. Current models engage users in therapeutic conversations, with many using “rule-based” scripted responses and predefined dialog structures [6]. This technology differs from generative AI chatbots, such as ChatGPT (OpenAI), which generate original content and provide lifelike interactions that closely imitate humans.

Generative AI can offer more empathetic emotional support in comparison to rule-based chatbots [4,7-9], making it more effective in counseling conversations and more acceptable to clients. A recent qualitative study exploring the efficacy of ChatGPT in supporting 24 individuals with mental health conditions found that more than 50% of participants found ChatGPT to be empathetic and 80% said the tool helped to manage symptoms [10]. However, participants also raised concerns regarding data privacy and the risk of personal data theft, uncertainty in the reliability of the information shared and the quality of training data, and a lack of understanding of cultural determinants of mental health.

Generative AI chatbots may also provide improved access to mental health information and psychoeducation. Preprint and peer-reviewed studies assessing the quality of psychoeducational information generated by ChatGPT on depression, anxiety, psychosis, and substance abuse found that it was accurate, clear, clinically useful, relevant, and empathetic [11-13]. Furthermore, studies have found generative AI chatbots to be effective in supporting adherence to psychological treatments [14] and delivering positive psychology interventions [15,16]. These studies indicate that generative AI chatbots may improve individuals’ access to information and basic therapeutic exercises, allowing clinicians to focus on more complex tasks, such as diagnosis and treatment.

Studies have identified a variety of uses of AI for practitioners, including research, documentation, administering psychological assessments, clinic management, and triaging [5,17-19]. Generative AI could enhance these functions by offering faster processing speeds, more comprehensive client histories, and higher empathy [20,21]. However, like rule-based AI models, studies have found generative AI chatbots are prone to mistakes, with one study finding that ChatGPT included incorrect information in 36% of client documents in a medical setting [21]. Another study evaluated ChatGPT’s performance in conducting mental health assessments and found that, while responses for simple cases were acceptable, recommendations for more complex cases were inappropriate and potentially harmful [22]. These findings highlight the risks associated with AI, including hallucinations and misinterpretations.

Generative AI may also be capable of more sophisticated functions, such as recommending diagnoses and treatment options. Bartal et al [23] found that ChatGPT was able to accurately identify posttraumatic stress disorder (PTSD) following childbirth by analyzing individuals’ birth stories, achieving high sensitivity (85%) and specificity (75%), aligning with commonly accepted benchmarks of reliability [24,25]. Similarly, another case study demonstrated that ChatGPT can identify treatment-resistant schizophrenia and suggest appropriate treatment options [26]. Despite these innovative applications, some argue that there is a need for significant improvement before AI is adopted in mental health care [27].

Clinicians appear cautious about the use of AI and feel more education and training are required [28,29]. In a systematic review of AI in mental health care, Rogan et al [28] found that most studies highlighted a need for clinician training, emphasizing that “AI will only be useful if clinicians understand and feel comfortable using it”. Similarly, in a survey of 138 American psychiatrists, Blease et al [29] found that 90% of participants agreed that clinicians need more support to understand generative AI. Qualitative responses were mixed, with some participants expressing uncertainty and concerns relating to potential harm and others overestimating the readiness of generative AI to deliver clinical tasks.

While practitioners have expressed a need for more training and support, many may already be using AI tools for internal purposes. Blease et al [29] found that more than half of psychiatrists surveyed had used generative AI chatbots to answer clinical questions. A survey of 86 Australian mental health professionals found that 43% had used AI and that ChatGPT was the most commonly used tool [19]. Likewise, a systematic review preprint on the use of generative AI in health care found that the technology is “heavily applied” for documentation, research, and administration among medical practitioners [30].

Research examining practitioners’ use of generative AI chatbots with clients is more limited. One study found strong support among clinicians to use chatbots with clients, with 80% of participants reporting they would be either very likely (24%) or somewhat likely (56%) to recommend AI mental health chatbots to clients within the next 5 years [31]. Participants may have been more supportive as they were asked to consider their future likelihood and, therefore, the potential for evolution of both the technology and their understanding.

### Objective

This study aims to explore which functions of generative AI chatbots mental health practitioners think are most useful, and to what extent they would recommend them to clients. In addition, this study will investigate the impact of exposure to a generative AI chatbot on participants’ likelihood of recommending chatbots to clients.

This study uses both quantitative and qualitative data to address 3 research questions (RQ) aligned with corresponding hypotheses. These RQs address key gaps in the literature, including limited insight into which functions practitioners prefer, a lack of empirical data on their willingness to recommend generative AI chatbots to clients, and minimal understanding of how exposure to the technology might influence those attitudes.

RQ1 explores practitioners’ views on the most useful functions of generative AI chatbots in mental health care. It is hypothesized that functions such as generating case notes (hypothesis 1), supporting session planning (hypothesis 2), and identifying literature (hypothesis 3) will be prioritized. Previous research suggests clinicians favor using AI for internal tasks like documentation and research [29,30].

RQ2 examines the likelihood of mental health practitioners recommending a generative AI chatbot to clients. It is hypothesized (hypothesis 4) that fewer than 50% will do so, as practitioners typically have low technology literacy [28,29,32].

RQ3 investigates whether practitioners are more likely to recommend an AI chatbot after a demonstration, with the prediction (hypothesis 5) that exposure to a demo will significantly increase their likelihood to recommend it. Research indicates that familiarity with health care technologies improves clinician adoption [33].

## Method

### Participants

Participants were recruited using a purposive, convenience sampling method. Digital flyers were distributed on forums including the Australian Psychological Society (APS), Australian Association of Psychologists Inc (AAPi), through Melbourne University channels, and with friends and family. Individuals who were not working in a client-facing, mental health role were excluded from the study.

In total, 23 individuals participated in the study, and all were working in Australia. Sample demographics are presented in Table 1.

**Table 1.** Sample demographic information (N=23).

Demographic variables	Participants (N=23)
Gender, n (%)	
Men	6 (26)
Women	17 (74)
Age (years), mean (SD)	39.39 (16.20)
Clinical role, n (%)	
Psychologist (including provisional)	10 (30)
Counselor or psychotherapist	6 (26)
Mental health social worker	4 (17)
Psychiatrist	2 (9)
Mental health support worker	1 (4)
Ethnicity, n (%)	
Caucasian or European	15 (65)
Asian	5 (22)
Mixed ethnicity	3 (13)
Area of work, n (%)	
Private sector	8 (35)
Public sector	9 (39)
Mixed	6 (26)
Years of work experience, mean (SD)	10.78 (14.31)

### Procedure

The study team devised interview questions (Multimedia Appendix 1) to explore practitioners’ perspectives on generative AI chatbots in mental health care. The interviews were of 45 minutes and conducted over Zoom (Zoom Video Communications, Inc.) between May and August 2024. Open-ended, Likert-scale, and ranking questions were used to collect both qualitative and quantitative data. Interviews were recorded and transcribed using AI software, Notta (Notta Inc), to support qualitative analysis.

### Uses of Generative AI Chatbots in Mental Health Care

Participants were asked to select their top 3 “most useful” functions of generative AI chatbots from a list of 11 options and explain their reasoning. The uses were:

1. Generating case notes
2. Support with session planning
3. Client triaging
4. Client onboarding processes
5. Administering psychological assessments
6. Supporting therapist-directed exercises
7. Supporting self-directed exercises
8. Symptom tracking and monitoring
9. Identifying and suggesting literature relevant to a client’s profile
10. Psychoeducation and socialization to therapeutic models
11. Counseling

An option to select “Other” was offered and subsequently removed from the analysis as it was not selected by any participants.

### Likelihood of Recommending a Chatbot to a Client

Participants were asked, “How likely are you to recommend a chatbot to a client?” on a Likert scale of 1 (Highly unlikely) to 4 (Highly likely). This question was asked both before (T1) and after (T2) viewing a demonstration of a generative AI chatbot.

The demonstration was an 11-minute screen recording of a generative AI chatbot that had been built using GPT-4o (OpenAI) for the purposes of this research. During the demonstration, a fictional client (“Saman”) interacted with the chatbot to illustrate a range of use cases, from triaging, psychological assessment, clinic onboarding, counseling, and completing a gratitude exercise. The demonstration also showed Saman’s fictional therapist using the chatbot to generate case notes, identify literature relevant to Saman’s profile, and plan future sessions (Multimedia Appendix 2).

Although the demonstration featured a chatbot built using GPT-4o, participants were asked to rate and reflect on generative AI chatbots for mental health support more generally, rather than the specific tool presented. Only the text-based conversational capacity of GPT-4o was used, with safety filters (mechanisms that monitor and restrict the model’s output to reduce risk of harm), and real-time context (allowing the chatbot to access external data and information) active by default. These features were not explicitly evaluated but were considered reflective of current generative AI chatbot capabilities.

### Data Analysis

#### Quantitative

##### RQ1: Uses of Generative AI Chatbots

Data were analyzed to determine the proportion of participants who selected each use and plot the SE. To test statistical hypotheses (hypothesis 1, hypothesis 2, and hypothesis 3), 3 binomial tests were conducted to assess whether specific uses were selected at greater than chance levels.

##### RQ2: Likelihood of Recommending a Chatbot to a Client

A binomial test was conducted to test hypothesis 4 and analyze whether less than 50% of participants would be likely to recommend a chatbot. A Bayesian binomial test was conducted to evaluate the strength of the evidence in favor of the alternative hypothesis.

##### RQ3: Increase in Likelihood of Recommending a Chatbot After a Demonstration

To test hypothesis 5, a paired-samples *t* test was conducted to measure whether there was a significant difference in the mean likelihood of recommending a chatbot before (T1) and after (T2) the demonstration. A Bayesian paired samples *t* test assessed the strength of the evidence in favor of the alternative hypothesis (a significant increase in recommendation likelihood from T1 to T2).

### Qualitative

A thematic analysis was conducted following Braun and Clarke’s [34] 6-phase framework. Analysis began with familiarization, where transcripts were reviewed to identify initial ideas. Themes were then generated based on recurring concepts, iterated, and refined to ensure they captured core insights. Finally, qualitative themes were considered alongside the quantitative results to identify key research findings. Where participants are quoted, a reference is provided with their participant identification number (#), gender (men or women), and age.

### Ethical Considerations

All participants provided informed consent and no compensation was offered. Participants were informed in advance that participation was voluntary, and they could withdraw at any time without consequence. Data were stored securely, and participant confidentiality was secured by anonymizing all data. Ethics approval was obtained from the University of Melbourne Human Research Ethics Committee (#28479).

## Results

### Overview of Qualitative Themes

**Figure 1.**
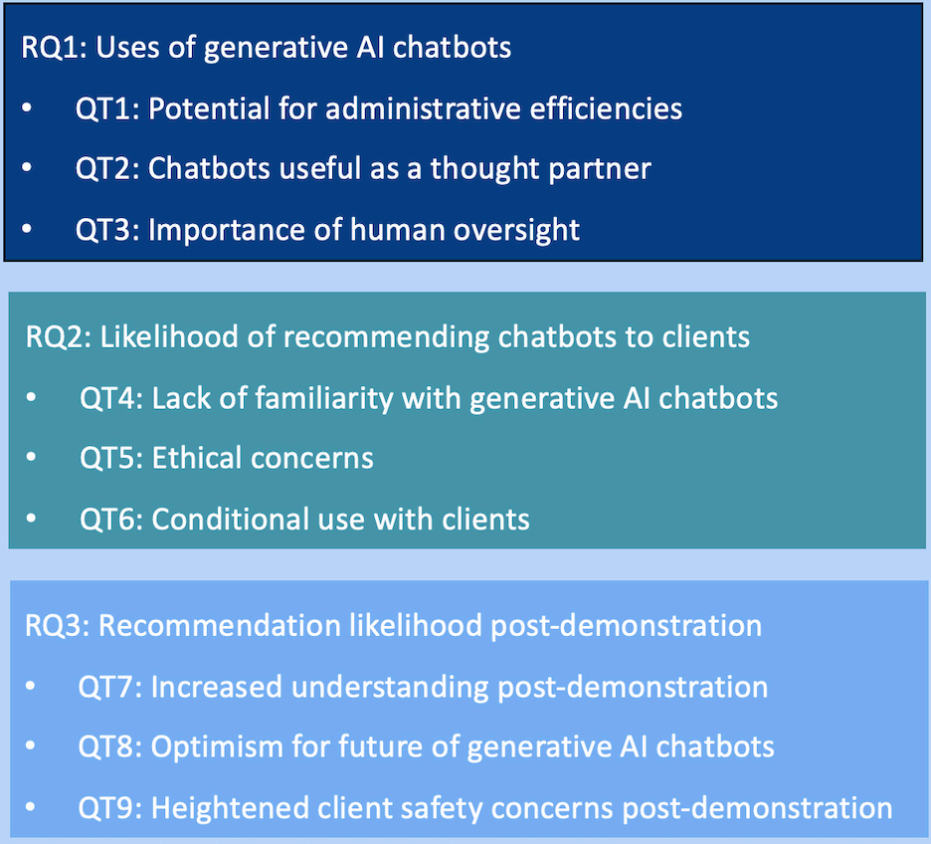
Summary of qualitative themes.

Thematic analysis produced 9 qualitative themes (QTs), each aligned to a particular RQ, as shown in Figure 1. These themes are reported in further detail throughout the following section.

### RQ1: Uses of Generative AI Chatbots

**Figure 2.**
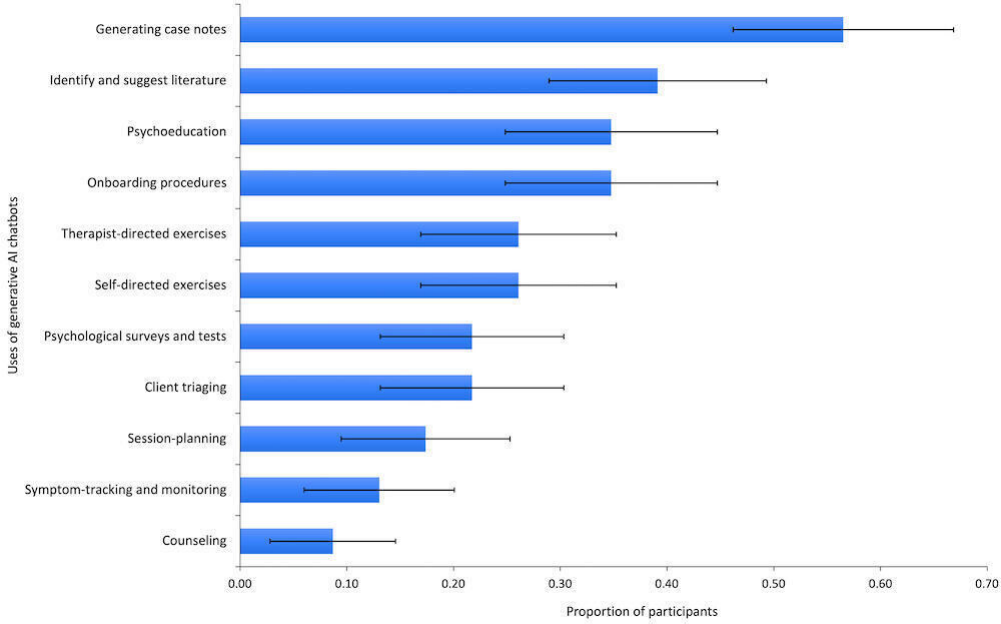
Proportion of participants selecting each use of generative AI chatbots. Error bars represent the SE of the proportion of participants selecting each use.

Proportions of participants who selected each use in their top 3 are presented in Figure 2.

A binomial test was conducted to assess whether Generating case notes was selected in participants’ top 3 choices at greater than chance levels. The chance probability was calculated at 0.25, based on the probability of a participant selecting an option, acknowledging that they had 3 choices and could not select the same option twice (*P*(selected)=1 - [1- 1/11] x [1 - 1/10] x [1- 1/9]). The results were significant, *P*=.001, indicating that Generating case notes was selected at greater than chance levels. A binomial test indicated that Support with session planning was not selected in participants’ top 3 choices at greater than chance levels (0.25; *P*=.86). A binomial test indicated that Identifying literature and Suggesting literature was not selected in participants’ top 3 choices at greater than chance levels (0.25; *P*=.10).

#### QT1: Potential for Administrative Efficiencies

Qualitative analysis indicated that participants value the potential of generative AI chatbots to reduce time spent writing case notes. One participant explained:

*Writing clinical notes, that takes up probably more time in therapists’ days than they would like... having a process to be able to. dump your very verbatim notes from a session and have it spit out a very succinct summary of the mental state formulation. That would be awesome*

[#20, F, 29].

Another participant emphasized that using a chatbot to document case notes would enable them to spend more time with clients,

*Generating case notes strikes me as just a time-consuming and aversive activity… you save yourself time to spend more time with clients and less time on admin*

[#4, M, 42].

#### QT2: Chatbots Useful as a Thought Partner

Qualitative responses indicated that participants saw the potential of generative AI chatbots to assist with session planning by providing new ideas, though it wasn’t seen as a necessity. One participant stated,

*For help with session planning I feel like well it’s good with just reflecting on ideas and you know talk about things that you maybe haven’t thought about*

[#2, F, 27].

Another explained,

*I mean, you could do it without it, but it’s always helpful to just to maybe get another perspective or consider something else*

[#17, F, 35].

#### QT3: Importance of Human Oversight

While qualitative responses indicated that participants hoped generative AI chatbots might enhance their research, many stressed the importance of maintaining human oversight. One participant explained:

*[Identifying literature] is something that we do digitally anyway and if we’re able to customize a search to a client specific profile, that might make the search better. And then obviously, the clinician can still have the final say on that, but it would be helpful*

[#9, F, 24].

Several participants raised concerns around accuracy, with one participant remarking that,

*It would be great to have a reliable source that can give us the latest research in what is best practice CBT [Cognitive Behavioral Therapy] for OCD [Obsessive Compulsive Disorder]... [ChatGPT] just never seems quite accurate*

[#11, F, 25].

Another explained,

*I know that there’s been a lot of speculation around finding accurate references… I would always, you know, [use ChatGPT] in addition to my own search as well, just to be sure*

[#2, F, 27].

### RQ2: Likelihood of Recommending a Chatbot to a Client

**Figure 3.**
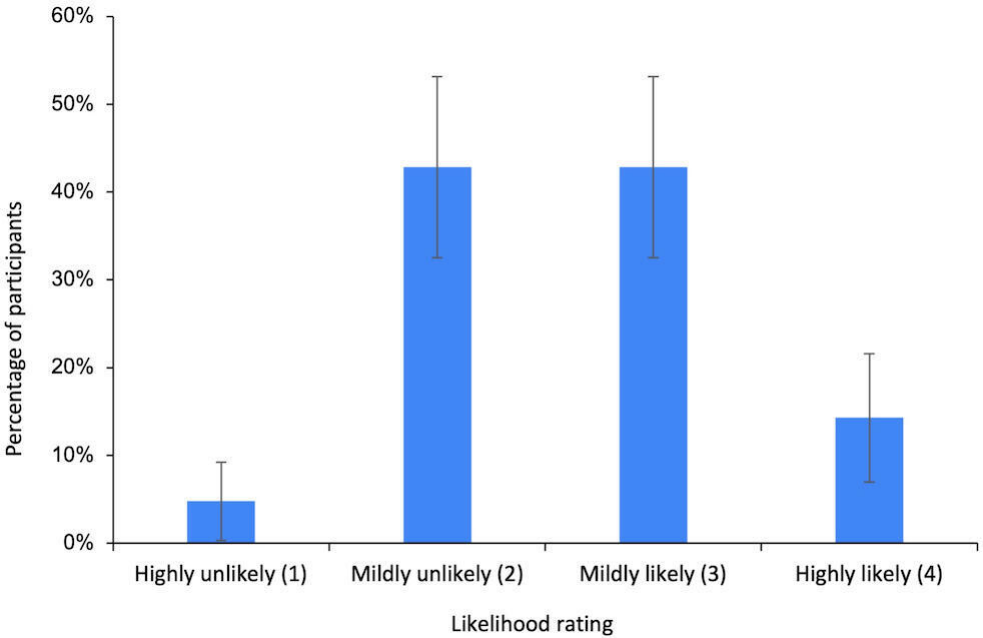
Distribution of participants’ likelihood of recommending a generative AI chatbot (n = 22). Error bars represent the SE of the percentage of participants selecting each rating.

The distribution of participants’ likelihood of recommending a chatbot to a client at T2 is presented in Figure 3. Data from T2 (postdemonstration) was used for the analysis, as it best reflected participants’ informed perspectives of generative AI chatbots. One participant responded, “I don’t know,” and their data were excluded from the quantitative analysis.

A binomial test was conducted to determine whether participants would be likely to recommend a generative AI chatbot to a client (defined as a response of 3 or 4 on the 4-point Likert scale). The proportion of participants likely to recommend was 55% (12/22), which was not significantly different from the 50% threshold (*P*=.74; odds ratio 1.20 [95% CI 0.47-3.17]). A Bayesian binomial test provided moderate evidence for the null hypothesis that more than 50% of participants would be likely to recommend a chatbot (BF_01_=5.24).

#### QT4: Lack of Familiarity With Generative AI Chatbots

Most participants were not familiar with generative AI chatbots in mental health care. One participant noted,

*I don’t feel like I understand the market well enough yet to recommend it to clients. I would need more experience and a chance to evaluate different models before trusting them for client use*

[#4, M, 42].

Others explained that they simply “don’t know enough about it”. During introductory questions, only one participant reported having experience using an AI chatbot for mental health support for themselves or with a client.

#### QT5: Ethical Concerns

Ethical concerns were frequently cited. One participant explained,

*I don’t think that it would be prudent or appropriate to recommend [chatbots] to clients just the same way that we wouldn’t recommend unapproved treatments that we can’t be fully confident in to clients because then we basically have liability*

[#9, F, 24].

Another commented,

*Privacy would be my main concern. How do we manage to keep client information confidential if we’re using it for note purposes?*

[#20, F, 29].

#### QT6: Conditional Use With Clients

For many participants, their likelihood of recommending a chatbot was dependent on the type of client. One participant stated,

*I do see a use for it as an emergency point of contact, but for anything beyond that, especially involving complex risk, I wouldn’t feel comfortable recommending it*

[#9, F, 24].

Another explained that their etiological formulation would inform whether a chatbot would be appropriate:

*A lot of people’s addictions and mood disorders are born out of childhood trauma, and I think there is also a certain percentage of those who are* *enduring* *attachment wounds. And that’s where AI is not going to be able to help*

[#12, F, 66].

Others suggested their likelihood of recommending would be based on age, with one participant explaining,

*It depends on the demographic. Maybe if it was a young person, I would. But in my practice, I don’t actually come across many young people. So personally, probably no, because most of my demographics are older*

[#6, F, 54].

### RQ3: Change in Likelihood of Recommending a Chatbot to a Client After the Demonstration

A one-tailed paired samples *t* test was conducted to assess whether there was a statistically significant increase in the likelihood of recommending a chatbot from T1 (mean 2.09 [SD 1.07]) to T2 (mean 2.64 [SD 0.79]). Results were significant, *t*_21_=3.20; *P*=.002, with a mean difference of 0.55. The effect size (*d*=0.68) was medium-to-large [35], suggesting a meaningful increase in recommendation likelihood.

A Bayesian paired samples *t* test provided strong evidence in favor of the alternative hypothesis (*r*=0.707; BF_10_=20.08), indicating that participants were more likely to recommend a chatbot after the demonstration (T2) compared to before (T1).

#### QT7: Increased Understanding Postdemonstration

Qualitative analysis suggested that the demonstration improved participants’ understanding of how generative AI chatbots can be used in mental health care. One participant stated,

*I have a better sense of the additional value that AI can provide particularly around screening and triage*

[#4, M, 42].

Another stated that, “It gave me new ideas on onboarding and case notes” (#1, F, 25). Some participants were satisfied with how the chatbot performed client-facing tasks such as counseling, with one participant stating,

*I was quite happy with how [the chatbot] dealt with everything in terms of like when it realized that the issues were quite severe and that it didn’t jump to try to diagnose*

[#2, F, 27].

#### QT8: Optimism for Future of Generative AI Chatbots

After the demonstration, several participants expressed optimism about the potential of generative AI. One participant remarked,

*I imagine as the technology gets better and we’re able to further program them to work in a really safe and diligent way around this sort of stuff, it probably will work quite smoothly and reliably*

[#7, F, 27].

One participant likened the introduction of AI chatbots to the evolution of telephone counseling, while another stated:

*I know a lot of people firmly believe that AI will never be able to deliver that human touch. And I just think that I just think they’re delusional, frankly, I think, I think they need to… realize what’s coming*

[#4, M, 42]

#### QT9: Heightened Client Safety Concerns Postdemonstration

Of the 10 participants who reported an increased likelihood of recommending a chatbot, 4 indicated that the demonstration had heightened their concerns regarding client safety. One participant stated,

*I was spooked by how the AI responded when the person expressed suicidality… it slightly ups my sense of the risk based on current technology*

[#5, F, 41].

Another commented,

*The [user] was feeling a bit flat and all he was left with was a phone number, like rather than ‘I will put you through to Lifeline right now’, for example, just leaving risk like that, that’s probably not helpful*

[#8, F, 58].

## Discussion

### Principal Findings

This study found that practitioners appear to prefer administrative uses of generative AI chatbots, such as generating case notes, and are uncertain about recommending chatbots to clients. While exposure to a demonstration may increase the likelihood of recommending a chatbot, concerns regarding client safety and risk persist.

The first hypothesis (hypothesis 1) was supported. Generating case notes was selected as the most useful function of generative AI chatbots at greater than chance levels. The second (hypothesis 2) and third (hypothesis 3) hypotheses were not supported. Support with session planning and Identifying and suggesting literature were not selected at greater than chance levels. The fourth hypothesis (hypothesis 4), that less than 50% of participants would be likely to recommend a chatbot to a client, was not supported. The fifth hypothesis (hypothesis 5) was supported; participants were significantly more likely to recommend a chatbot to a client postdemonstration, and Bayesian analysis provided strong evidence for this increase.

### Practitioners Prefer Administrative Uses of Generative AI Chatbots

The results indicate a preference among practitioners for using generative AI chatbots for simple, administrative tasks. Quantitative and qualitative data highlight that practitioners see value in generative AI chatbots for drafting and summarizing case notes. Participants expressed enthusiasm for the potential administrative efficiencies that using a chatbot would allow more time to focus on client-facing work. This aligns with previous studies where practitioners reported using generative AI tools for documentation and research [19,29,30]. This preference is also evident in the influx of AI-based tools for case note documentation in the market, such as Heidi (Heidi Health), Upheal (Upheal), and Autonotes (Autonotes AI, LLC) [36-38].

There is; however, some nuance in the types of administrative tasks practitioners are willing to use generative AI chatbots for. While descriptive data showed that Identifying and suggesting literature was the second most frequently selected use, it was not selected at greater than chance levels. Similarly, there was a lack of support for Session planning. Participants emphasized the importance of human oversight, indicating a lack of trust in generative AI. This aligns with findings from Blease et al [29], where psychiatrists were cautious of using generative AI for tasks that require clinical expertise and raised concerns about accuracy. As such, while practitioners may not be willing to delegate more complex administrative tasks to generative AI chatbots, this study suggests that there is potential for the technology to be applied in a supporting capacity.

### Practitioners Are Uncertain About Recommending Generative AI Chatbots to Clients

This study found no clear preference for or against recommending a chatbot to clients, with uncertainty evident in both quantitative and qualitative results. Both the frequentist and Bayesian analyses reflected statistical uncertainty regarding the true proportion of participants who were likely to recommend a chatbot to clients. Qualitative analysis highlighted several ethical concerns, ranging from professional liability to confidentiality and data security, and a lack of familiarity with the technology. This aligns with previous research, which found that practitioners often harbor ethical concerns and lack education on the appropriate use of generative AI tools in practice [29,32].

This uncertainty contrasts with Sweeney et al [31], where 80% of practitioners were likely to recommend a chatbot to a client. This variance may be explained by the difference in how questions were phrased. In Sweeney et al [31] study, participants were asked whether they would recommend a chatbot “in the next five years,” allowing practitioners to consider how the technology, and their understanding of it, may evolve. In contrast, the present study focused on immediate likelihood, which may have biased participants toward considering current limitations. Participants also expressed optimism about the future of generative AI, suggesting they may have been more supportive if asked to consider their likelihood of recommending a chatbot in the future.

### Practitioners’ Use of Generative AI Chatbots May Be Conditional on the Client

A unique insight from this study was practitioners’ support for conditional use of generative AI chatbots, depending on factors unique to the client. Qualitative responses indicated that participants were more inclined to recommend chatbots for low-risk, less complex cases (ie, stress, sleep issues, and life transitions) compared to high-risk (ie, suicidal intent) or trauma-related cases. The existing literature on the appropriate client profiles for generative AI chatbots presents mixed views. Some propose that AI is appropriate for use with clients with complex clinical histories, on the basis that the technology can analyze and track data on, for example, genetic markers and behavioral patterns [39]. Others suggest that AI chatbots may be most appropriate in prevention and psychoeducation in low-risk populations [40]. Ultimately, like any mental health treatment, the success of generative AI tools will depend not only on the technology, but also on its fit for the client.

### Exposure Increases Recommendation Likelihood and May Heighten Client Safety Concerns

This study found that participants’ likelihood of recommending a generative AI chatbot to a client increased significantly after a demonstration. Qualitative insights provided depth to the quantitative results, as participants explained that the demonstration had increased their understanding of how chatbots can be applied in mental health care. This result aligns with earlier findings of a positive correlation between health care practitioners’ knowledge of generative AI chatbots and positive attitudes toward the technology [41]. Similarly, previous studies have found that negative perceptions of AI among mental health professionals may be due to minimal exposure [29,42].

Alternatively, participants’ increased recommendation likelihood postdemonstration may be reflective of novelty bias, where individuals tend to exhibit increased enthusiasm toward a new or unfamiliar technology or idea [43]. Qualitative data suggests that for 22 out of 23 participants (96%), the demonstration was the first time they had seen a generative AI chatbot used in a mental health setting. In a similar study by Liu et al [16], it was observed that participants responded more positively to a chatbot trained to deliver positive psychology interventions during initial interactions.

Surprisingly, despite the quantitative increase in recommendation likelihood, qualitative responses indicated that the demonstration heightened client safety concerns for some participants. Of the ten participants who reported an increased recommendation likelihood, 4 described being more concerned about the risks. This suggests that a greater understanding of the associated risks of using generative AI chatbots may play a role in adoption among practitioners. This aligns with results from Zhang et al [42], where participants described the importance of understanding the risks of AI to “clinician buy-in” and adoption of the technology in practice. While an examination of potential risks was beyond the scope of this study, this finding underscores the importance of providing evidence-based, objective education to practitioners.

### Limitations and Future Directions for Research

Several limitations of this study should be addressed. First, convenience sampling may have introduced selection bias, as individuals who were more supportive of AI technologies may have been more likely to participate, leading to an overrepresentation of positive attitudes. Future studies should apply random sampling methods to reduce selection bias, ensure representativeness, and improve external validity [44]. In addition, participants may have been aware that the interviewers created the chatbot demonstration, introducing demand characteristics and potential bias toward more favorable responses. The use of independent researchers to administer questionnaires may assist in mitigating potential bias in future research.

A primary focus of this research was to gather in-depth qualitative data to allow for greater exploration of practitioners’ perspectives. Due to project constraints and the time required of participants, the study sample size was small (N=23). While this number is appropriate for qualitative thematic research [45], it limited the statistical power and reliability of the quantitative analyses [46]. No a priori power calculation was conducted, as the study was primarily exploratory and qualitatively focused. In addition, the sample was Australian, predominantly female (17/23, 74%) and mostly psychologists and counselors (13/23, 56%). Caution should be exercised when generalizing these findings to other health systems or sociocultural contexts. In the future, researchers should recruit larger, more representative samples in different cultural contexts, while still maintaining the qualitative analysis that is crucial for understanding the nuanced perspectives of practitioners.

Participants were asked to rate their likelihood of recommending a chatbot to a client on a 4-point Likert scale. While the absence of a neutral midpoint encouraged participants to share their perspectives, it may have forced practitioners to choose a position that they did not fully support.

This study did not consider recent developments in generative AI chatbot technology, such as voice recognition or more sophisticated emotional detection capabilities [47]. Researchers may consider partnering with AI developers to test emerging technologies to ensure that studies remain relevant, while also allowing for practitioner and user feedback on tools before they are released to market.

### Practical Implications

For practitioners, the findings suggest that generative AI chatbots could be valuable in alleviating their administrative burden. However, the study also highlights the need for careful consideration as to how AI is applied and the need for practitioners to improve their understanding of AI chatbots, particularly as their clients may already be using them [22].

Educational institutions and industry associations play an important role in ensuring practitioners have appropriate AI skills and literacy and are prepared to deliver services in a field where technology is playing an increasingly important role [48]. Practical, evidence-based guidelines for the use of AI tools should address concerns expressed in this study regarding liability, client safety, and ethics, while also enabling effective use of technology to support positive mental health outcomes. Practitioners should also be informed of emerging research so as to help validate their decision-making and points of view.

For AI developers, insights from this study may support iteration and refinement of generative AI chatbots for mental health care. For example, developers should ensure practitioners’ concerns regarding client safety, data security, and accuracy are addressed in AI tools and service agreements. This study also highlights the need for developers to engage practitioners, as well as users, throughout the design process to support safe and effective technology implementation.

### Conclusion

Generative AI chatbots have significant potential to play an important role in the delivery of mental health care. In the immediate term, their greatest value may be in administrative, nonclient-facing tasks, to alleviate practitioner workload and allow greater attention to be directed to therapeutic work. This study highlighted the complexity and nuance of practitioners’ perspectives on generative AI chatbots. While there is interest and optimism, there is a lack of familiarity with AI tools and uncertainty regarding client-facing uses. Furthermore, exposure to AI technology may play an important role in supporting adoption among practitioners.

Future research should continue to explore practitioners’ views on administrative and client-facing uses with larger, more representative samples to enhance the reliability and generalizability of findings. It would also be beneficial to explore hybrid models and the benefits of therapist-AI collaboration. Such evidence could inform objective guidelines to allow practitioners to make informed decisions on their use of generative AI chatbots. Ultimately, this study provides valuable contributions as to “how,” rather than simply “whether,” generative AI chatbots may be integrated into mental health care, guiding more thoughtful, stakeholder-informed implementation.

## Supplementary material

10.2196/71065 Multimedia Appendix 1 Interview questions and interviewer script.

10.2196/71065 Multimedia Appendix 2 Demonstration transcript and link.

## Abbreviations

AAPi: Australian Association of Psychologists Inc
AI: artificial intelligence
APS: Australian Psychological Society
PTSD: posttraumatic stress disorder
QT: qualitative theme
RQ: research question
T1: recommending a chatbot before demonstration
T2: recommending a chatbot after demonstration

## References

[1] Kupcova I, Danisovic L, Klein M, Harsanyi S (2023). Effects of the COVID-19 pandemic on mental health, anxiety, and depression. BMC Psychol.

[2] (2024). Mental health services. Australian Institute of Health and Welfare.

[3] Mental Health Australia Report to the nation 2023. https://mhaustralia.org/sites/default/files/docs/report_to_the_nation_2023.pdf.

[4] Fitzpatrick KK, Darcy A, Vierhile M (2017). Delivering cognitive behavior therapy to young adults with symptoms of depression and anxiety using a fully automated conversational agent (Woebot): a randomized controlled trial. JMIR Ment Health.

[5] Vaidyam AN, Wisniewski H, Halamka JD, Kashavan MS, Torous JB (2019). Chatbots and conversational agents in mental health: a review of the psychiatric landscape. Can J Psychiatry.

[6] Maglogiannis I, Iliadis L, Pimenidis E (2020). IFIP Advances in Information and Communication Technology (AIAI 2020 Conference Proceedings).

[7] Bird T, Mansell W, Wright J, Gaffney H, Tai S (2018). Manage your life online: a web-based randomized controlled trial evaluating the effectiveness of a problem-solving intervention in a student sample. Behav Cogn Psychother.

[8] Enos G (2023). Digital mental health agent for youths shows results similar to therapy group. Mental Health Weekly.

[9] Fulmer R, Joerin A, Gentile B, Lakerink L, Rauws M (2018). Using psychological artificial intelligence (Tess) to relieve symptoms of depression and anxiety: randomized controlled trial. JMIR Ment Health.

[10] Alanezi F (2024). Assessing the effectiveness of ChatGPT in delivering mental health support: a qualitative study. J Multidiscip Healthc.

[11] Coşkun AB, Elmaoğlu E, Buran C, Yüzer Alsaç S Integration of Chatgpt and E-Health Literacy: Opportunities, Challenges, and a Look Towards the Future. Journal of Health Reports and Technology.

[12] Yilanli M, McKay I, Jackson DI, Sezgin E (2024). Large language models for individualized psychoeducational tools for psychosis: a cross-sectional study. medRxiv.

[13] Maurya RK, Montesinos S, Bogomaz M, DeDiego AC (2025). Assessing the use of ChatGPT as a psychoeducational tool for mental health practice. Couns and Psychother Res.

[14] Yasukawa S, Tanaka T, Yamane K (2024). A chatbot to improve adherence to internet-based cognitive–behavioural therapy among workers with subthreshold depression: a randomised controlled trial. BMJ Ment Health.

[15] Wang Y, Li S (2024). Tech vs. tradition: ChatGPT and mindfulness in enhancing older adults’ emotional health. Behav Sci (Basel).

[16] Liu I, Liu F, Xiao Y, Huang Y, Wu S, Ni S (2025). Investigating the key success factors of chatbot-based positive psychology intervention with retrieval- and generative pre-trained transformer (GPT)-based chatbots. International Journal of Human–Computer Interaction.

[17] Davenport T, Kalakota R (2019). The potential for artificial intelligence in healthcare. Future Healthc J.

[18] Townsend BA, Plant KL, Hodge VJ, Ashaolu OT, Calinescu R (2023). Medical practitioner perspectives on AI in emergency triage. Front Digit Health.

[19] Cross S, Bell I, Nicholas J (2024). Use of AI in mental health care: community and mental health professionals survey. JMIR Ment Health.

[20] Sharma A, Lin IW, Miner AS, Atkins DC, Althoff T (2023). Human–AI collaboration enables more empathic conversations in text-based peer-to-peer mental health support. Nat Mach Intell.

[21] Baker HP, Dwyer E, Kalidoss S, Hynes K, Wolf J, Strelzow JA (2024). ChatGPT’s ability to assist with clinical documentation: a randomized controlled trial. J Am Acad Orthop Surg.

[22] Dergaa I, Fekih-Romdhane F, Hallit S (2023). ChatGPT is not ready yet for use in providing mental health assessment and interventions. Front Psychiatry.

[23] Bartal A, Jagodnik KM, Chan SJ, Dekel S (2024). AI and narrative embeddings detect PTSD following childbirth via birth stories. Sci Rep.

[24] Guest R, Tran Y, Gopinath B, Cameron ID, Craig A (2018). Prevalence and psychometric screening for the detection of major depressive disorder and post-traumatic stress disorder in adults injured in a motor vehicle crash who are engaged in compensation. BMC Psychol.

[25] Maurer DM, Raymond TJ, Davis BN (2018). Depression: screening and diagnosis. Am Fam Physician.

[26] Galido PV, Butala S, Chakerian M, Agustines D (2023). A case study demonstrating applications of ChatGPT in the clinical management of treatment-resistant schizophrenia. Cureus.

[27] Pandya A, Lodha P, Ganatra A (2024). Is ChatGPT ready to change mental healthcare? Challenges and considerations: a reality-check. Front Hum Dyn.

[28] Rogan J, Bucci S, Firth J (2024). Health care professionals’ views on the use of passive sensing, AI, and machine learning in mental health care: systematic review with meta-synthesis. JMIR Ment Health.

[29] Blease C, Worthen A, Torous J (2024). Psychiatrists’ experiences and opinions of generative artificial intelligence in mental healthcare: An online mixed methods survey. Psychiatry Res.

[30] Wang L, Wan Z, Ni C (2024). A systematic review of chatgpt and other conversational large language models in healthcare. Health Informatics.

[31] Sweeney C, Potts C, Ennis E (2021). Can chatbots help support a person’s mental health? Perceptions and views from mental healthcare professionals and experts. ACM Trans Comput Healthcare.

[32] Blease C, Kharko A, Annoni M, Gaab J, Locher C (2021). Machine learning in clinical psychology and psychotherapy education: a mixed methods pilot survey of postgraduate students at a Swiss University. Front Public Health.

[33] Torous J, Bucci S, Bell IH (2021). The growing field of digital psychiatry: current evidence and the future of apps, social media, chatbots, and virtual reality. World Psychiatry.

[34] Braun V, Clarke V (2006). Using thematic analysis in psychology. Qual Res Psychol.

[35] Cohen J (1988). Statistical Power Analysis for the Behavioral Sciences.

[36] Upheal.

[37] Heidi Health.

[38] Autonotes.

[39] Olawade DB, Wada OZ, Odetayo A, David-Olawade AC, Asaolu F, Eberhardt J (2024). Enhancing mental health with artificial intelligence: current trends and future prospects. Journal of Medicine, Surgery, and Public Health.

[40] Ettman CK, Galea S (2023). The potential influence of AI on population mental health. JMIR Ment Health.

[41] Li Y, Li Z (2024). Knowledge, attitude, and practices regarding ChatGPT among health care professionals. Am J Manag Care.

[42] Zhang M, Scandiffio J, Younus S (2023). The adoption of AI in mental health care-perspectives from mental health professionals: qualitative descriptive study. JMIR Form Res.

[43] Mirnig A, Gaertner M, Meschtscherjakov A, Tscheligi M (2020). Blinded by novelty: a reflection on participant curiosity and novelty in automated vehicle studies based on experiences from the field.

[44] Andrade C (2021). The inconvenient truth about convenience and purposive samples. Indian J Psychol Med.

[45] Guest G, Bunce A, Johnson L (2006). How many interviews are enough? An experiment with data saturation and variability. Field methods.

[46] Button KS, Ioannidis JPA, Mokrysz C (2013). Power failure: why small sample size undermines the reliability of neuroscience. Nat Rev Neurosci.

[47] OpenAI (2023). ChatGPT can now see, hear, and speak OpenAI website.

[48] Rajaei A (2024). Teaching in the age of AI/ChatGPT in mental-health-related fields. The Family Journal.

