# Supplementary material for: Practitioner Perspectives on the Uses of Generative AI Chatbots in Mental Health Care: Mixed Methods Study
Source: JMIR Hum Factors. 2025 Sep 16;12:e71065. doi: 10.2196/71065 (PMC12440320; doi:10.2196/71065)
Supplement: Multimedia Appendix 1 [file humanfactors-v12-e71065-s001.docx]

**Multimedia Appendix 1**

**Interview Script**

**Interviewer Introduction:**

As you will be aware, the purpose of this study is to understand practitioners’ perspectives on the use of artificial intelligence, or AI, chatbots in mental health care. In this interview, I’m going to ask you a series of questions and will be gathering both qualitative and quantitative data through the use of scales. I will be typing notes throughout the interview, but I hope this isn’t too distracting. We will also be recording this interview, however, the recording will only be used for note-taking purposes and will not be shared.

In this interview, we will be talking about AI and, specifically, Generative AI which you might be familiar with through using Chat GPT or similar tools. We will be discussing generative AI mental health chatbots, which are designed to engage in conversations with users about mental health. Generative AI helps mental health chatbots understand what people say about their feelings and thoughts, and then respond in a way that feels like a natural conversation. The chatbots can therefore simulate empathetic responses and provide personalised advice, rather than using technology to select from pre-programmed or “canned” responses.

Please let me know if you have any questions at this stage, or feel free to ask for clarification as we go.

**Demographic Questions:**

- Age, gender, ethnicity, nationality
- Type of clinician/role
- Years of experience in mental health care
- Area of work (public sector/private practice/both)

**Substantive Interview Questions:**

1. What experience do you have with chatbots? Which chatbots have you used and in what contexts?
2. Are you aware of the existence of chatbots to support mental health and administer mental health therapies? If so, which bots are you aware of?

Now I’d like to ask your opinion on the use of AI for therapeutic applications

1. What are the benefits of using an AI chatbot for mental health support?
2. What are your concerns about using an AI chatbot for mental health support?
3. Do you think the benefits of using AI technology in a therapeutic setting outweigh the risks? Rate on the below scale, or say “I don’t know”: 1 = strongly disagree, 2 = mildly disagree, 3 = mildly agree, and 4 = strongly agree.  Why or why not?
4. How likely are you to recommend an AI chatbot for mental health support to a client?

Rate on the below scale, or say “I don’t know”: 1 = highly unlikely, 2 = mildly unlikely, 3 = mildly likely, and 4 = highly likely. Why or why not?

1. If an AI chatbot therapist could adequately provide/administer the following actions or therapies, which would be most useful?

Please choose your top 3 and explain why you chose them:

- Client triaging
- Onboarding procedures
- Generating case notes
- Help with session-planning
- Identify and suggest literature relevant to the specific client’s profile
- Administration of psychological surveys and tests
- Symptom tracking and monitoring
- Psychoeducation and socialisation to therapeutic models
- Counselling
- Supporting completion of therapist-directed exercises (e.g. CBT tasks including exposure, and thought challenging)
- Supporting completion of self-directed exercises (e.g. mindfulness, gratitude journaling)
- Other (please specify)

1. Please rate the following statements on a scale of 1 to 4, or say “I don’t know”: 1 = strongly disagree, 2 = mildly disagree, 3 = mildly agree, and 4 = strongly agree.

- AI chatbots will be widely accessible at low cost to people worldwide, making mental health support more universally available.
- An AI chatbot will lack contextual or personal understanding of their clients’ culture, situations, histories, and experiences.
- AI chatbots will facilitate early intervention and preventative care
- It would be beneficial for AI chatbot to offer intensive therapies between sessions
- The ability of the AI chatbot to intervene in response to the client’s activity on social media would benefit some clients
- AI chatbots will misdiagnose or misidentify the client’s issues
- AI chatbots will provide incorrect or inappropriate treatment recommendations
- If clients perceive the AI chatbot to be free of judgment they would be more likely to engage in therapy
- The lack of regulatory oversight of AI-based care poses a risk to clients
- The ability for AI chatbots to provide multilingual support will be beneficial
- The ability of an AI chatbot to be immediately updated with the latest research findings will be beneficial
- An AI chatbot will lack empathy or the ability to create a connection with the client
- An AI chatbot can be available 24/7, which will be beneficial for clients.
- AI chatbots pose a data security risk and/or a risk to the privacy of the client
- An AI chatbot would avoid bias and stereotyping in conversations with clients
- It would be helpful for an AI chatbot to remind and assist clients in doing their homework between sessions, e.g. offering reminders to do meditation exercises or fill out thought diaries
- AI chatbots will suggest harmful or illegal activities to clients
- There is a risk that the clients will over-rely on the bot for support

1. Do you think that an AI chatbot therapist would be able to adequately respond to or manage a crisis? For example, suicidal intentions, risk of harm to others or admission of illegal activity. Why or why not?

*[Demonstration of the bot’s capabilities, transcript of demonstration shown at Appendix 2.]*

1. How has your understanding changed after seeing this demonstration?
2. Given what we have spoken about, do you think the benefits of using AI technology in a therapeutic setting outweigh the risks? Rate on the below scale: 1 = strongly disagree, 2 = mildly disagree, 3 = mildly agree, and 4 = strongly agree. Why or why not?
3. Given what we have spoken about, how likely are you to recommend an AI chatbot for mental health support to a client? Rate on the below scale: 1 = highly unlikely, 2 = mildly unlikely, 3 = mildly likely, and 4 = highly likely.
4. Do you have any other comments you would like to share?
